# Design and analysis of virtual impedance control scheme based on MESOGI for improving harmonic sharing of nonlinear loads

**DOI:** 10.1038/s41598-024-62739-z

**Published:** 2024-06-11

**Authors:** Abdelhammid Kherbachi, Ahmed Bendib, Aissa Chouder, Hafiz Ahmed, Mohamed Benbouzid, Saad Motahhir

**Affiliations:** 1 Centre de Développement des Energies Renouvelables, CDER, Route de l’Observatoire, B.P. 62, 16340 Bouzaréah, Algiers, Algeria; 2 SET Laboratory, Electronics Department, Blida University, BP 270, Blida, Algeria; 3 Electrical Engineering Laboratory (LGE), University Mohamed Boudiaf of Msila, BP166, 28000 M’Sila, Algeria; 4 Nuclear Advanced Manufacturing Research Centre, The University of Sheffield, Rutherford Way, Infinity Pk Wy, Derby, DE73 5SS UK; 5 University of Brest, UMR CNRS 6027 IRDL, 29238 Brest, France; 6 ENSA Sidi Mohammed Ben Abdellah University, 30000 Fez, Morocco

**Keywords:** DC offset, Harmonic power-sharing, Modeling, Multiple Enhanced Second-Order Generalized Integrator (MESOGI), Nonlinear load, Virtual impedance (VI), Electrical and electronic engineering, Energy science and technology

## Abstract

Under the presence of nonlinear load, the most existing virtual impedance (VI) methods-based control solution performs poorly in reactive power sharing among droop-operated VSIs in microgrids (MGs). This may be due to the involved estimation techniques for extracting the current harmonics at selected frequencies, which suffer from either poor accuracy of the harmonic estimation and/or the effect of DC offset in the measurements. Such an issue may affect the performance of the virtual impedance control, hence, the system stability. To bridge this gap, the implementation of the virtual impedance based on multiple enhanced second-order generalized integrator (MESOGI) suitable for harmonics and DC-offset estimation/rejection, is proposed in this paper. The MESOGI can offer an accurate estimation of the current quadrature components free from DC offset at selected frequencies, required to implement the virtual impedance control. Therefore, it makes the designed virtual impedance-based control scheme robust to voltage distortions, immune to DC disturbance, and capable of sharing properly the power harmonics. As a result, this may contribute to improving the reactive and harmonic power-sharing between droop-controlled VSIs within an islanded MG. The modeling of the MESOGI scheme and its performance investigation is carried out. In addition, the mathematical model of the implemented virtual impedance is derived. Further, analysis based on the obtained model of the equivalent output impedance including virtual impedance is established to study its effect. Simulation and experimental tests are performed to prove the effectiveness of the control proposal in improving the reactive power sharing under nonlinear load operating conditions.

## Introduction

Microgrid based on distributed energy resources is seen as a promising topology to meet the customer's electricity demands^1–3^. One of the major advantages of these MGs is the capability to be connected to the main grid or operate autonomously. During autonomous mode operation, the main objective is to guarantee active and reactive power-sharing between distributed generation units. This is the responsibility of the droop control strategy-based primary control, which can offer real and reactive power-sharing through adjusting the DGs' frequency and voltage by using only local measurements^4–8^. However, this control strategy has a major drawback, which is the poor reactive power-sharing due to the DG feeders’ impedance mismatch^9,10^. Further, under nonlinear load operating conditions, the accuracy of the power-sharing may be severely affected and it is difficult to share the power harmonics. To overcome this issue, the virtual impedance concept is adopted to mitigate the effect of the line impedance mismatch, thereby enhancing reactive power-sharing.

Various approaches have been reported in the literature for the design and implementation of the virtual output impedance^11–29^. For instance, the simplest method adopted to implement the virtual impedance has been presented in^11^, which uses the multiplication of the current derivate by a predefined virtual inductance to obtain the expected output signal. Indeed, this method offers ease of implementation but due to the absence of the filtering process, the performance of this method can be severely affected by the current distortions. To deal with this issue, a low-pass filter (LPF) is introduced into the virtual impedance to mitigate undesirable current distortions^12^. However, the use of an LPF may slow down the transient performance of the system control. The SOGI-FLL method, which can offer high filtering capability and good transient response, is an effective solution that has been considered for the implementation of the virtual impedance^22^. In these methods, the output estimates of the SOGI-FLL, *v*_*α*_ and *v*_*β*_ components, are used to achieve the output of the virtual impedance. Such a method has less sensitivity to the output current noise, achieves output free from harmonic distortion, and contributes to enhancing reactive power-sharing. Despite these benefits, the presence of the DC component in the SOGI input results in an undesirable shift in the estimated quadrature component, therefore deteriorating the performance of the virtual impedance-based control scheme. In this regard, the TOGI algorithm, which can provide the rejection capability of the DC component and its effects, has been adopted in^23^, to implement the virtual impedance. Although this method has dealt with the effect of the DC offset, it faces a significant issue which is the inability to share the power harmonic under nonlinear load operating conditions. Therefore, harmonic power-sharing cannot be guaranteed when the DG units feed nonlinear loads. To this end, the implementation of the virtual impedance by using the MSOGI strategy has been developed in^24^, to regulate the virtual impedance at the fundamental frequency and selected harmonic frequencies. In addition, in^13^ and^25^, the implementation of the virtual impedance based on MDSOGI is proposed considering the power-sharing at the fundamental frequency and selected harmonic frequencies. It is worth mentioning that, these two methods can offer the estimation of the desired components with high precision, which is assured by rejecting all the other components from the one to be extracted. Despite, the reported methods having offered the sharing of power harmonic, they still suffer from the issue of the DC disturbance, which may affect MSOGI and MDSOGI estimation proprieties, therefore degrading the control performance. To bridge this gap, a virtual impedance implementation based on band-pass filters (BPFs) for extracting the expected components at selected harmonic frequencies is adopted^26^. Indeed, this method has dealt with both the harmonic power-sharing and the adverse effect of the DC offset. However, the accuracy of the estimated expected components at the fundamental and selected harmonic frequencies by using BPF is questionable compared to the MSOGI. As a result, proper reactive and harmonic power-sharing between parallelized VSIs can not be guaranteed.

To address this issue, the implementation of the virtual impedance control scheme based on MESOGI for droop-controlled single-phase VSIs is proposed in this paper. The proposed method-based power-sharing control scheme aims to achieve accurate active and reactive power sharing among parallel inverters considering nonlinear load conditions and DC disturbance. The main contributions made in the present paper to reach this objective are:

- An improved virtual impedance control scheme based on the MESOGI suitable for harmonics and DC-offset estimation/rejection is developed. The MESOGI method can achieve an accurate estimation of the current quadrature components at fundamental and selected frequencies and free from DC offset from a highly distorted current. These estimated components are used to obtain the output voltage of the virtual impedance. Therefore, a robust virtual impedance control scheme and immune to voltage distortions and DC component effect is achieved; thereby a control scheme with reactive and harmonic power-sharing performance enhancement is expected.
- A modeling procedure is developed to obtain the mathematical model of the MESOGI structure. By using this model, an investigation of the MESOGI estimation performance is established to show the capability of the MESOGI to estimate the quadrature components at fundamental and selected frequencies with high precision.
- The model of the MESOGI-based virtual impedance implementation is derived. Then, the mathematical expression of the equivalent output virtual impedance, including the implemented virtual impedance and inner control dynamics, is also extracted and the effect of the virtual impedance is studied.

Simulations and experimental tests are performed to validate the effectiveness of the proposed control schemes in achieving accurate power-sharing among paralleled DG units even under nonlinear load operating conditions and different distortions.

## Proposed control scheme

**Figure 1:**
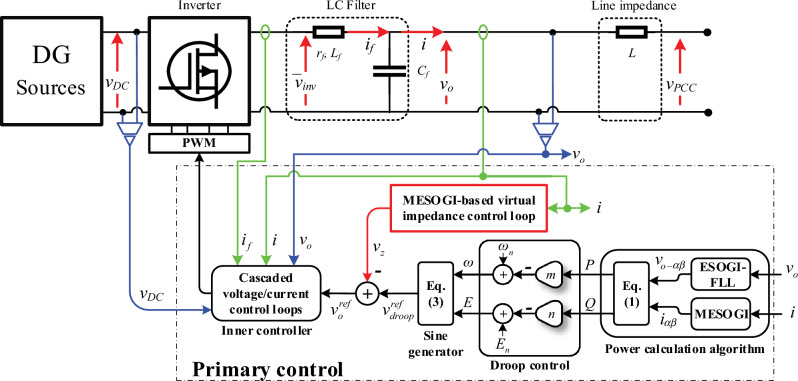
Proposed control scheme for a single-phase inverter.

Figure 1 shows the proposed control scheme for droop-controlled single-phase VSI connected to the point of common coupling (PCC); where the load is linked; via an LC filter and a line impedance. This control scheme comprises; a droop control method, a proposed virtual impedance control unit, and an inner voltage controller.

1. The droop controller is in charge of ensuring active and reactive power-sharing between paralleled VSIs. This control scheme includes a power calculation unit based on MESOGI-FLL, droop method, and sinusoidal signal generator. The power calculation is in charge of computing the average real and reactive power in the *αβ*-frame by using the direct and quadrature components of the inverter output current and voltage;$i_{\alpha \beta }$ and $v_{\alpha \beta }$; estimated by the MESOGI and ESOGI-FLL, respectively. Accordingly, the expression of the average real and reactive power, *P* and *Q*, can be defined as follows^30^:
  1 $$\begin{gathered} P = \frac{1}{2}\left( {\hat{v}_{o - \alpha } \hat{i}_{\alpha } + \hat{v}_{o - \beta } \hat{i}_{\beta } } \right) \hfill \\ Q = \frac{1}{2}\left( {\hat{v}_{o - \beta } \hat{i}_{\alpha } - \hat{v}_{o - \alpha } \hat{i}_{\beta } } \right) \hfill \\ \end{gathered}$$

The droop method uses the computed real and reactive power to generate the frequency and amplitude references of the inverter output voltage based on the *P/f* and *Q/V* characteristics given as follows:

2 $$\begin{gathered} \omega = \omega_{n} - mP \hfill \\ E = E_{n} - nQ \hfill \\ \end{gathered}$$

where *m* and *n* are the droop gains. It is worth mentioning that these characteristics define the case where the line impedance is considered purely inductive.

The sine generator exploits the produced references, *f* and *E*, to provide the reference of the inverter output voltage, which can be defined as follows:

3 $$v_{droop}^{ref} (t) = E \times \sin \left( {\omega \times t} \right)$$

2. The proposed virtual impedance control unit based on MESOGI-FLL is integrated to enhance reactive power and harmonic sharing. The output voltage, *v*_*z*_, of this control unit, is subtracted from the droop controller’s output voltage reference to produce the new voltage reference *v*_*o*_ defined by (4). More details about this control unit are given in the following section.
  4 $$v_{o} (t) = E \times \sin \left( {\omega \times t} \right) - v_{z} \left( t \right)$$
3. The double-loop inner controller is intended for adjusting the inverter output voltage to its reference. This control scheme consists of an outer voltage feedforward proportional-integral (PI) controller for the control of the capacitor voltage, and an inner current proportional controller for adjusting the current of the LC filter impedance to its reference. The schematic diagram of these control loops is given in “Appendix A”, and more details can be found in^31^.

## Proposed virtual output impedance based on MESOGI

### Description of the proposed structure

**Figure 2:**
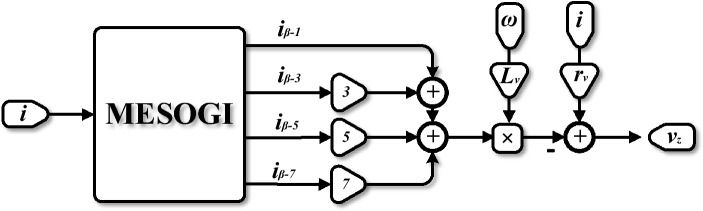
Block diagram of virtual-impedance implementation based on MESOGI.

Figure 2 depicts the structure of the proposed virtual impedance implemented based on the MESOGI method suitable for harmonic and DC offset estimation/rejection. In this structure, the MESOGI is integrated to estimate the quadrature fundamental component (*i*_*β-1*_) and its versions corresponding to 3rd, 5th, and 7th harmonics (*i*_*β-3*_, *i*_*β-5*_, and *i*_*β-7*_) from the actual output current, *i*, of the inverter. Note that in our purpose only the three first harmonics; i.e., 3rd, 5th, 7th; are considered.

The sum of the estimated quadrature components; multiplied by a gain *h* corresponding to the harmonic’s order; is multiplied by the virtual inductor *L*_*v*_. The resulting signal is subtracted from the actual output current multiplied by the virtual resistor, *r*_*v*_, to get the output voltage of the virtual impedance, *v*_*z*_ expressed as:

5 $$v_{z} \left( t \right) = r_{v} i\left( t \right) - L_{v} \omega \left( {i_{\beta - 1} + 3i_{\beta - 3} + 5i_{\beta - 5} + 7i_{\beta - 7} } \right)$$

where *s* is the Laplace variable and *ω* denotes the FLL estimated frequency from the output voltage (for more details about the FLL scheme refer to^32^).

The MESOGI can provide an accurate estimation of the quadrature fundamental and harmonic components free from DC offset even for highly distorted current^33^. Also, according to (5), the implemented virtual impedance takes into consideration these components for obtaining its output. Therefore, it is expected to achieve the possibility of harmonic current sharing and improvement of the reactive power balance as well, especially in the case of supplying nonlinear loads. To better clarify the performance enhancement of the proposed MESOGI-based virtual impedance implementation, analyses are carried out of what came. Further, as the implemented virtual impedance depends on the outputs of the MESOGI, i.e., *i*_*β−1*_, *i*_*β−3*_, *i*_*β−5*,_ and *i*_*β−7*_, the modeling procedure for obtaining the dynamic model of the MESOGI regarding estimation of its outputs, is first established.

### Modeling of MESOGI

#### Structure of the MESOGI

**Figure 3:**
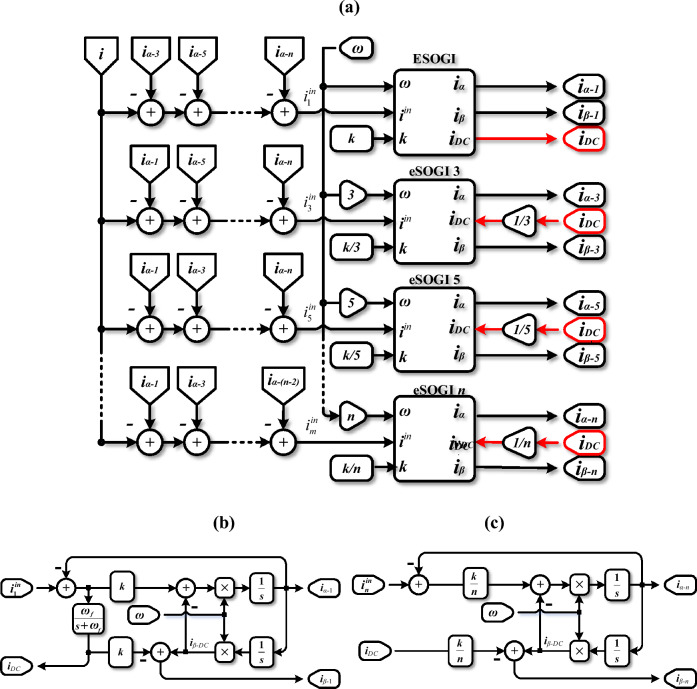
Architecture of; (**a**) MESOGI, (**b**) ESOGI, and (**c**) eSOGI methods.

The structure of the proposed MESOGI employed to precisely estimate the components of the output current under extreme distortions (DC-offset disturbance and nonlinear load), is shown in Fig. 3a. This proposed structure consists of ESOGI and *n* eSOGI, suitable for DC component rejection, connected in parallel, which are given in Fig. 3b and c, respectively. The ESOGI unit is formed by incorporating a DC component estimation/rejection block into the conventional SOGI (see Fig. 3b). Whereas, the eSOGI unit introduces the DC component estimated by the ESOGI, as an input to be canceled from the quadrature component. These units provide total rejection of the DC component as well as its effect on the output components^32^. Note that to facilitate the description of the proposed MESOGI and mathematical development, let us consider that ESOGI refers to both ESOGI and eSOGI. Each ESOGI, in the proposed structure, is an adaptive filter tuned to a specified center frequency, which is obtained by multiplying the fundamental frequency estimated using the FLL by a coefficient that specifies the assigned harmonic’s order. In addition, each ESOGI *k* gain is divided by such a coefficient corresponding to the harmonic order to ensure uniform transient response times across all ESOGI blocks (see “Appendix B”). The input current of each ESOGI unit is determined by subtracting the in-phase output components of the other ESOGI units from the actual input current. Therefore, each SOGI’s input current undergoes a purification process after a transient phase, which removes the harmonic components estimated by the other ESOGI units, effectively rejecting harmonic distortions at its output. As a result, accurate estimation of the current fundamental and harmonic components free from DC offset; which are required for the implementation of the virtual impedance; is expected.

#### Mathematical modeling

The objective of this part is to present the modeling procedure that allows the derivation of the transfer functions describing the dynamics of the MESOGI output estimates. These functions relate the estimated outputs of the MESOGI to its actual input current *i*.

According to Fig. 3b and c, the expression of the output signals *i*_*α−n*_, *i*_*β−n*_, and *i*_*DC*_ of an ESOGI to its clean input current $i_{n}^{in}$ (*n* = 1, 3, …, *n*) can be obtained as:

6 $$\left\{ \begin{gathered} i_{\alpha - n} = G_{\alpha - n} i_{n}^{in} \hfill \\ i_{\beta - n} = G_{\beta - n} i_{n}^{in} - \left( {k/n} \right)i_{DC} \hfill \\ i_{DC} = G_{DC} \left( {i_{1}^{in} - i_{\alpha - 1} } \right) \hfill \\ \end{gathered} \right.$$

where *i*_*α-n*_ and *i*_*β-n*_ are the in-phase and in-quadrature components corresponding to *n* order harmonic, $i_{1}^{in}$ and *i*_*α−1*_ are the input current fundamental component and its direct version, *i*_*DC*_ is the estimated DC component of the single-phase input current, and *G*_*α-n*_, *G*_*β-n*_, and *G*_*DC*_ are the ESOGI’s transfer functions expressed as:

7 $$\left\{ \begin{gathered} G_{\alpha - n} = \frac{{i_{\alpha - n} }}{{i_{n}^{in} }} = \frac{k\omega s}{{s^{2} + k\omega s + n^{2} \omega^{2} }} \hfill \\ G_{\beta - n} = \frac{{i_{\beta - n - DC} }}{{i_{n}^{in} }} = \frac{{kn\omega^{2} }}{{s^{2} + k\omega s + n^{2} \omega^{2} }} \hfill \\ G_{DC} = \frac{{i_{DC} }}{{i_{1}^{in} - i_{\alpha - 1} }} = \frac{{\omega_{f} }}{{s + \omega_{f} }} \hfill \\ \end{gathered} \right.$$

where *ω*_*f*_ is the LPF cutoff frequency.

On the other hand, from Fig. 3a, the cleaned input current of each ESOGI can be defined as a function of the actual input current as follows:

8 $$i_{j}^{in} = i_{{}} - \sum\limits_{\begin{subarray}{l} p = 0 \\ i = 2p + 1 \\ i \ne j \end{subarray} }^{n} {i_{\alpha - i} }$$

This equation can be written in a matrix form as follows:

9 $$\left[ {\begin{array}{*{20}c} {i_{1} } \\ {i_{3} } \\ {i_{5} } \\ \vdots \\ {i_{n} } \\ \end{array} } \right]^{in} = - \left[ {\begin{array}{*{20}c} 0 & 1 & 1 & \cdots & 1 \\ 1 & 0 & 1 & \cdots & 1 \\ 1 & 1 & 0 & \cdots & 1 \\ \vdots & \vdots & 1 & \ddots & \vdots \\ 1 & 1 & 1 & \cdots & 0 \\ \end{array} } \right]\left[ {\begin{array}{*{20}c} {i_{\alpha - 1} } \\ {i_{\alpha - 3} } \\ {i_{\alpha - 5} } \\ \vdots \\ {i_{\alpha - n} } \\ \end{array} } \right] + \left[ {\begin{array}{*{20}c} 1 \\ 1 \\ 1 \\ \vdots \\ 1 \\ \end{array} } \right]i_{{}}$$

Considering (8), the transfer functions of the ESOGI’s output components can be expressed in a matrix form as outlined below.

*a. i*_*α−n*_ that is in phase with the input current $i_{n}^{in}$ of each ESOGI unit ${\varvec{i}}_{{\varvec{n}}}^{{{\varvec{in}}}}$

10 $$\left[ {\begin{array}{*{20}c} {i_{\alpha - 1} } \\ {i_{\alpha - 3} } \\ {i_{\alpha - 5} } \\ \vdots \\ {i_{\alpha - n} } \\ \end{array} } \right] = \left[ {\begin{array}{*{20}c} {G_{\alpha - 1} } & 0 & 0 & \cdots & 0 \\ 0 & {G_{\alpha - 3} } & 0 & \cdots & 0 \\ 0 & 0 & {G_{\alpha - 5} } & \cdots & 0 \\ \vdots & \vdots & \vdots & \ddots & \vdots \\ 0 & 0 & 0 & \cdots & {G_{\alpha - n} } \\ \end{array} } \right]\left[ {\begin{array}{*{20}c} {i_{1} } \\ {i_{3} } \\ {i_{5} } \\ \vdots \\ {i_{n} } \\ \end{array} } \right]^{in}$$

*b. i*_*β−n*_ that is in-quadrature phase with the input current $i_{n}^{in}$ of each ESOGI unit

11 $$\left[ {\begin{array}{*{20}c} {i_{\beta - 1} } \\ {i_{\beta - 3} } \\ {i_{\beta - 5} } \\ \vdots \\ {i_{\beta - n} } \\ \end{array} } \right] = \left[ {\begin{array}{*{20}c} {G_{\beta - 1} } & 0 & 0 & \cdots & 0 \\ 0 & {G_{\beta - 3} } & 0 & \cdots & 0 \\ 0 & 0 & {G_{\beta - 5} } & \cdots & 0 \\ \vdots & \vdots & \vdots & \ddots & \vdots \\ 0 & 0 & 0 & \cdots & {G_{\beta - n} } \\ \end{array} } \right]\left[ {\begin{array}{*{20}c} {i_{1} } \\ {i_{3} } \\ {i_{5} } \\ \vdots \\ {i_{n} } \\ \end{array} } \right]^{in} - \left[ {\begin{array}{*{20}c} k \\ {k/3} \\ {k/5} \\ \vdots \\ {k/n} \\ \end{array} } \right]i_{DC}$$

*c. i*_*DC*_ is the DC component related to the input current $i_{n}^{in}$, and the output current components *i*_*α−n*_ of each ESOGI unit

12 $$i_{DC} = G_{DC} [\begin{array}{*{20}c} 1 & 0 & 0 & \cdots & 0 \\ \end{array} ]\left( {\left[ {\begin{array}{*{20}c} {i_{1} } \\ {i_{3} } \\ {i_{5} } \\ \vdots \\ {i_{n} } \\ \end{array} } \right]^{in} - \left[ {\begin{array}{*{20}c} {i_{\alpha - 1} } \\ {i_{\alpha - 3} } \\ {i_{\alpha - 5} } \\ \vdots \\ {i_{\alpha - n} } \\ \end{array} } \right]} \right)$$

Substituting (9) into (10)–(12), the expressions of the output current components estimated by the MESOGI scheme can be defined as follows:

- The direct current components *i*_*α−n*_
  13 $$\left[ {\begin{array}{*{20}c} {i_{\alpha - 1} } \\ {i_{\alpha - 3} } \\ {i_{\alpha - 5} } \\ \vdots \\ {i_{\alpha - n} } \\ \end{array} } \right] = \left[ {\begin{array}{*{20}c} 1 & {G_{\alpha - 1} } & {G_{\alpha - 1} } & \cdots & {G_{\alpha - 1} } \\ {G_{\alpha - 3} } & 1 & {G_{\alpha - 3} } & \cdots & {G_{\alpha - 3} } \\ {G_{\alpha - 5} } & {G_{\alpha - 5} } & 1 & \cdots & {G_{\alpha - 5} } \\ \vdots & \vdots & \vdots & \ddots & \vdots \\ {G_{\alpha - n} } & {G_{\alpha - n} } & {G_{\alpha - n} } & \cdots & 1 \\ \end{array} } \right]^{ - 1} \left[ {\begin{array}{*{20}c} {G_{\alpha - 1} } \\ {G_{\alpha - 3} } \\ {G_{\alpha - 5} } \\ \vdots \\ {G_{\alpha - n} } \\ \end{array} } \right]i_{{}}$$
- The orthogonal current components *i*_*β−n*_
  14 $$\left[ {\begin{array}{*{20}c} {i_{\beta - 1} } \\ {i_{\beta - 3} } \\ {i_{\beta - 5} } \\ \vdots \\ {i_{\beta - n} } \\ \end{array} } \right] = \left( \begin{gathered} - \left[ {\begin{array}{*{20}c} 0 & {G_{\beta - 1} } & {G_{\beta - 1} } & \cdots & {G_{\beta - 1} } \\ {G_{\beta - 3} } & 0 & {G_{\beta - 3} } & \cdots & {G_{\beta - 3} } \\ {G_{\beta - 5} } & {G_{\beta - 5} } & 0 & \cdots & {G_{\beta - 5} } \\ \vdots & \vdots & \vdots & \ddots & \vdots \\ {G_{\beta - n} } & {G_{\beta - n} } & {G_{\beta - n} } & \cdots & 0 \\ \end{array} } \right]\begin{array}{*{20}c} {\begin{array}{*{20}c} {} & \times \\ \end{array} } & {} \\ \end{array} \hfill \\ \left[ {\begin{array}{*{20}c} 1 & {G_{\alpha - 1} } & {G_{\alpha - 1} } & \cdots & {G_{\alpha - 1} } \\ {G_{\alpha - 3} } & 1 & {G_{\alpha - 3} } & \cdots & {G_{\alpha - 3} } \\ {G_{\alpha - 5} } & {G_{\alpha - 5} } & 1 & \cdots & {G_{\alpha - 5} } \\ \vdots & \vdots & \vdots & \ddots & \vdots \\ {G_{\alpha - n} } & {G_{\alpha - n} } & {G_{\alpha - n} } & \cdots & 1 \\ \end{array} } \right]^{ - 1} \left[ {\begin{array}{*{20}c} {G_{\alpha - 1} } \\ {G_{\alpha - 3} } \\ {G_{\alpha - 5} } \\ \vdots \\ {G_{\alpha - n} } \\ \end{array} } \right] + \left[ {\begin{array}{*{20}c} {G_{\beta - 1} } \\ {G_{\beta - 3} } \\ {G_{\beta - 5} } \\ \vdots \\ {G_{\beta - n} } \\ \end{array} } \right] \hfill \\ \end{gathered} \right)i_{o} - \left[ {\begin{array}{*{20}c} k \\ {k/3} \\ {k/5} \\ \vdots \\ {k/n} \\ \end{array} } \right]i_{DC}$$
- The DC output current component *i*_*DC*_
  15 $$i_{DC} = G_{DC} \left( {1 - \left[ {\begin{array}{*{20}c} 1 & 1 & 1 & \cdots & 1 \\ \end{array} } \right]\left[ {\begin{array}{*{20}c} 1 & {G_{\alpha - 1} } & {G_{\alpha - 1} } & \cdots & {G_{\alpha - 1} } \\ {G_{\alpha - 3} } & 1 & {G_{\alpha - 3} } & \cdots & {G_{\alpha - 3} } \\ {G_{\alpha - 5} } & {G_{\alpha - 5} } & 1 & \cdots & {G_{\alpha - 5} } \\ \vdots & \vdots & \vdots & \ddots & \vdots \\ {G_{\alpha - n} } & {G_{\alpha - n} } & {G_{\alpha - n} } & \cdots & 1 \\ \end{array} } \right]^{ - 1} \left[ {\begin{array}{*{20}c} {G_{\alpha - 1} } \\ {G_{\alpha - 3} } \\ {G_{\alpha - 5} } \\ \vdots \\ {G_{\alpha - n} } \\ \end{array} } \right]} \right)i_{{}}$$

Considering a simple case, in which the actual input current includes only the 3rd, 5th, and 7th order harmonics. Therefore, the expressions of the direct current components, *i*_*α−n*,_ and the orthogonal current components, *i*_*β−n*_, associated with the fundamental component, the 3rd, 5th, and 7th harmonics, and the DC component, *i*_*DC*_, of the MESOGI scheme are obtained as:

- For the output components [*i*_*α−*1_, *i*_*α−*3_, *i*_*α−*5_, *i*_*α−*7_]^*T*^
  16 $$\left[ {\begin{array}{*{20}c} {i_{\alpha - 1} } \\ {i_{\alpha - 3} } \\ {i_{\alpha - 5} } \\ {i_{\alpha - 7} } \\ \end{array} } \right] = \left[ {\begin{array}{*{20}c} {G_{BF.\alpha - 1} } \\ {G_{BF.\alpha - 3} } \\ {G_{BF.\alpha - 5} } \\ {G_{BF.\alpha - 7} } \\ \end{array} } \right]i_{{}}$$
- For the output components [*i*_*β−*1_, *i*_*β−*3_, *i*_*β−*5_, *i*_*β−*7_]^*T*^
  17 $$\left[ {\begin{array}{*{20}c} {i_{\beta - 1} } \\ {i_{\beta - 3} } \\ {i_{\beta - 5} } \\ {i_{\beta - 7} } \\ \end{array} } \right] = \left[ {\begin{array}{*{20}c} {G_{BF.\beta - 1} } \\ {G_{BF.\beta - 3} } \\ {G_{BF.\beta - 5} } \\ {G_{BF.\beta - 7} } \\ \end{array} } \right]i_{{}}$$
- For the output component *i*_*DC*_
  18 $$i_{DC} = G_{BF.DC} i$$
  where $G_{BF.\alpha - 1,3,5,7}$,$G_{BF.\beta - 1,3,5,7}$, and $G_{BF.DC}$ are the transfer functions that link the output in-phase, in-quadrature phase, and DC components to their input current, *i*, in which their expressions are provided in “Appendix C”.

**Figure 4:**
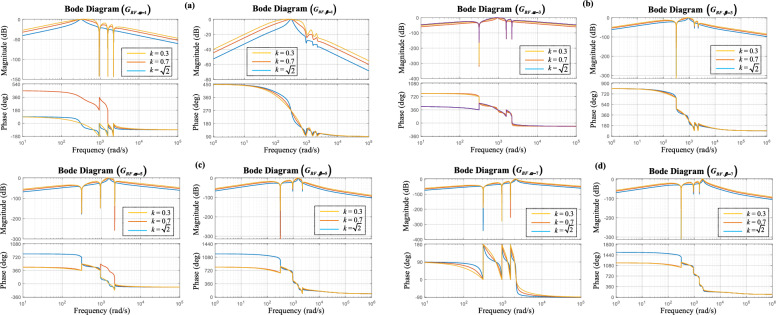
MESOGI’s frequency response in terms of the estimation of the direct and quadrature components (**a**) fundamental component, (**b**) 3rd harmonic, (**c**) 5th harmonic, and (**d**) 7th harmonic.

The bode plots of the closed-loop transfer functions $G_{BF.\alpha - 1,3,5,7}$ and $G_{BF.\beta - 1,3,5,7}$ of the 1st, 3rd, 5th, and 7th harmonics are depicted in Fig. 4a–d, respectively. The frequency responses of $G_{BF.\alpha - 1,3,5,7}$ and $G_{BF.\beta - 1,3,5,7}$ are shown for three distinct damping factor values; *k* = $\sqrt 2$, 0.7, and 0.3, while *ω* = 100π (rad/s). Observing these figures, it becomes evident that the transfer functions exhibit band-pass adaptive filter characteristics with bandwidths being contingent on *k*. In addition, as *k* increases, it results in reducing the filter bandwidth, consequently improving the sub- and low-harmonics filtering effectiveness. However, this effect decreases the transient response velocity of the estimation. Furthermore, it is noticed that the output components *i*_*α-*1_, *i*_*α-*3_, *i*_*α-*5_, and *i*_*α-*7_ exhibit matching amplitude and quadrature phase with the input current’s harmonics.

Consequently, the adopted MESOGI can guarantee accurate estimation of the in-phase and in-quadrature phase fundamental components. In addition, it can offer proper estimation of the 3rd, 5th, and 7th harmonics and high rejection capability of the DC component disturbance.

### Virtual impedance mathematical formulation and analysis

This part focuses mainly on studying the effect of the proposed virtual output impedance and highlighting its performance enhancement.

**Figure 5:**
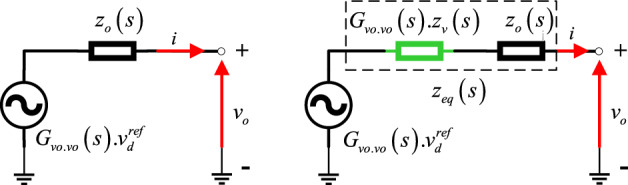
Thevenin equivalent circuit of a voltage-controlled VSI; (**a**) without, and (**b**) with virtual-output impedance*.*

Let mention that the key idea of introducing the virtual impedance in series to the inverter output impedance; as shown in Fig. 5b; is to increase the overall output impedance of the inverters with the same amount, to be highly inductive, highly resistive, or complex impedance. This will make the resulting equivalent output impedance higher compared to the line impedance and equal for all the DG units. As a result, the effect of the line impedance mismatch can be mitigated, and hence the reactive power-sharing can be improved. To better clarify this effect, an analysis based on a derived mathematical model of the equivalent output impedance including virtual impedance is conducted. It is worth mentioning that the derived model includes, the mathematical models of the output impedance with its associated control and virtual impedance, which are developed in what follows.

Figure 5 depicts the Thevenin equivalent circuit of a single-phase VSI with its associated inner controller. In this figure, the VSI with its inner controller is represented by a voltage-controlled source with an output impedance in series, with and without virtual output impedance as shown in Fig. 5a and b, respectively. Based on this figure, the expression of the output voltage *v*_*o*_ without virtual impedance and when considering the virtual impedance is given by (19) and (20), respectively.

19 $$v_{o} = \left[ {G_{vo - vo} \left( s \right)} \right].v_{droop}^{ref} - \left[ {z_{o} \left( s \right)} \right]i_{{}}$$

20 $$v_{o} = \left[ {G_{vo - vo} \left( s \right)} \right].v_{droop}^{ref} - \left[ {G_{vo - vo} \left( s \right).z_{v} \left( s \right) + z_{o} \left( s \right)} \right]i_{{}}$$

where *z*_*v*_ is the virtual impedance, *z*_*o*_ and *G*_*vo.vo*_ are the output impedance of the VSI including the inner control dynamics and the transfer function relating the output voltage to its output voltage reference of the inverter. The expressions of these transfer functions are given in “Appendix C”.

According to (20), the expression of the equivalent output impedance *z*_*G*_ including the virtual impedance can be concluded as follows:

21 $$z_{G} = G_{vo - vo} \left( s \right).z_{v} \left( s \right) + z_{o} \left( s \right)$$

in this equation, the expression of the virtual impedance, *z*_*v*_, should be defined.

Considering (5) and (17), the output of the MESOGI-based virtual impedance can be expressed, in matrix form, as:

22 $$v_{z} \left( s \right) = \left( {r_{v} - L_{v} \omega \left[ {\begin{array}{*{20}c} 1 & 3 & 5 & 7 \\ \end{array} } \right]\left[ {\begin{array}{*{20}c} {G_{BF.\beta - 1} } \\ {G_{BF.\beta - 3} } \\ {G_{BF.\beta - 5} } \\ {G_{BF.\beta - 7} } \\ \end{array} } \right]} \right)i_{{}}$$

Accordingly, the expression of the virtual output impedance, *z*_*v*_, can be derived as:

23 $$z_{v} \left( s \right) = r_{v} - L_{v} \omega \left[ {\begin{array}{*{20}c} 1 & 3 & 5 & 7 \\ \end{array} } \right]\left[ {\begin{array}{*{20}c} {G_{BF.\beta - 1} } \\ {G_{BF.\beta - 3} } \\ {G_{BF.\beta - 5} } \\ {G_{BF.\beta - 7} } \\ \end{array} } \right]$$

Therefore, considering (20) and (23), the nature of the output impedance of the inverter with virtual impedance can be modified by varying; the resistive and inductive parts; of the added virtual impedance.

**Figure 6:**
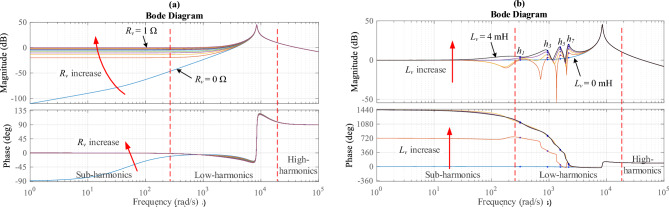
Output impedances’ frequency response, *z*_*o*_ and *z*_*G*_, for; (**a**) 0 Ω < *r*_*v*_ < 1 Ω, *L*_*v*_ = 0, and (**b**) 0 mH < *L*_*v*_ < 4 mH, *r*_*v*_ = 1 Ω.

The Bode diagram of the transfer functions of the output impedances *z*_*o*_ and *z*_*G*_ (with virtual-impedance; given by (23)) for an increase of the virtual resistance (*r*_*v*_) from 0 to 1 Ω and the virtual inductance (*L*_*v*_) from 0 to 4 mH are presented in Fig. 6. As can be seen in Fig. 6a, the equivalent output impedance *z*_*G*_ and the output impedance *z*_*o*_ have almost the same behavior for a small value of the virtual resistance *r*_*v*_. Whereas, when the value of the virtual resistance *r*_*v*_ increases the behavior of the equivalent output impedance, *z*_*G*_, changes from inductive to resistive with an increase in its magnitude at sub-frequencies (under fundamental frequency). Further, it can be noticed that the behavior of *z*_*G*_ at the high harmonics does not change no matter the value of *r*_*v*_. From Fig. 6b it can be observed that the magnitude of the equivalent output impedance *z*_*G*_ at the selected harmonics increases when increasing the value of the virtual inductance *L*_*v*_. Also, the behavior of the equivalent output impedance at these particular harmonics is complex (inductive/resistive). Further, it can be remarked that the behavior of *z*_*G*_ at the sub-frequencies is a variable resistor no matter how the *L*_*v*_ value changes. Furthermore, the equivalent output impedance (*z*_*G*_) behavior at the high harmonics does not change, no matter how the *L*_*v*_ value changes.

From these findings, one can conclude that the increase of the resistance part *r*_*v*_ increases the equivalent output impedance only at sub-harmonics, and this may lead to enhancement of sub-harmonics sharing (such as DC component). Whereas, the increase of the virtual inductance increases the equivalent output impedance at fundamental, and selective harmonics; 3rd, 5th, and 7th. This contributes to alleviating the effect of the line impedance mismatch at these harmonics and as a result, improves the harmonics sharing and reactive power balance.

## Simulation results

**Figure 7:**
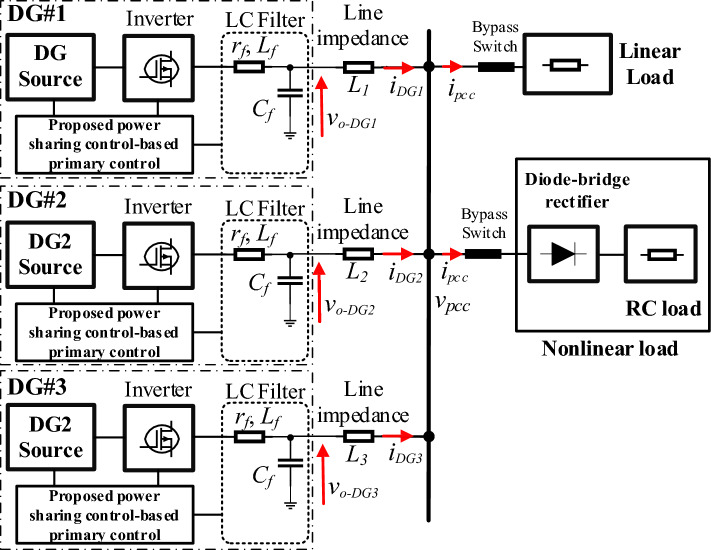
Simulation testbed consists of two DG units interfaced MG.

Simulation tests are carried out using MATLAB/Simulink to verify the effectiveness and robustness of the proposed virtual impedance implementation-based power-sharing control approach. The performance of the designed control scheme, in these tests, is investigated in response to linear and nonlinear loads sharing and upon changes in linear load as well. The simulation testbed is portrayed in Fig. 7, and it comprises three single-phase parallel-connected VSIs tied to a common AC bus via line impedances. The three inverters formed an autonomous MG that feeds a critical load. The linear load is an RL load with *R* = 20 Ω and *L* = 3 mH, while a full-bridge diode rectifier with an RC load; *R* = 200 Ω, *C* = 1000 μF; is used to model the nonlinear load. The power-sharing controller is similar to the one presented in Fig. 1 involving the proposed virtual impedance control scheme. The simulation system parameters are listed in Table 1.

**Table 1:** Parameters of the simulation study.

Parameter	Symbol	Unit	Value
Nominal voltage (RMS)	*E*_*n*_	V	220
Nominal frequency	*f*_*n*_	Hz	50
Switching frequency	*f*_*s*_	kHz	20
Simulation frequency	*f*_*e*_	MHz	1
DC voltage	*U*_*DC*_	V	495
Output filter capacitor	*C*	µF	23
Output filter inductor	*L, r*	mH, Ω	2, 1
DG #1 line impedance	*L*_*1*_	mH, Ω	1.5, 0.8
DG #2 line impedance	*L*_*2*_	mH, Ω	0.5, 0.8
DG #3 line impedance	*L*_*3*_	mH, Ω	1, 0.8
Virtual inductance	*L*_*v*_	mH	2.7
Virtual Resistance	*R*_*v*_	Ω	1
*P/ω* droop gain	*m*	rad/(W. s)	0.0005
*Q/V* droop gain	*n*	V/Var	0.001
P gain of the voltage controller	*k*_*pv*_	µF.rad/s	0.1839
I gain of the voltage controller	*k*_*pi*_	mH.rad/s	183.87
P gain of the current controller	*k*_*iv*_	mH.rad/s	6.2831

Sensors’ variance			
Parameter	Value		
Voltage sensors	22.5 V		
Current sensors	0.3 A		

Notice that to assess the system’s performance within a practical setting, Gaussian noise is deliberately added to both the sensors’ measurements and DC voltage sources.

**Figure 8:**
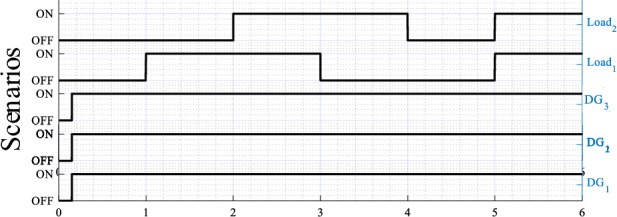
Plots of the applied scenarios in test 1.

**Figure 9:**
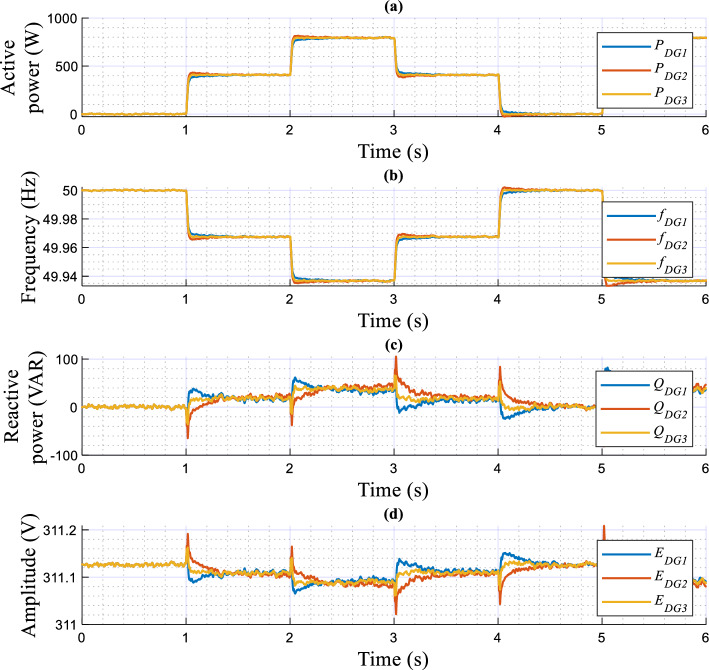
Simulation results of the proposed control strategy upon changes in the linear load.

**Figure 10:**
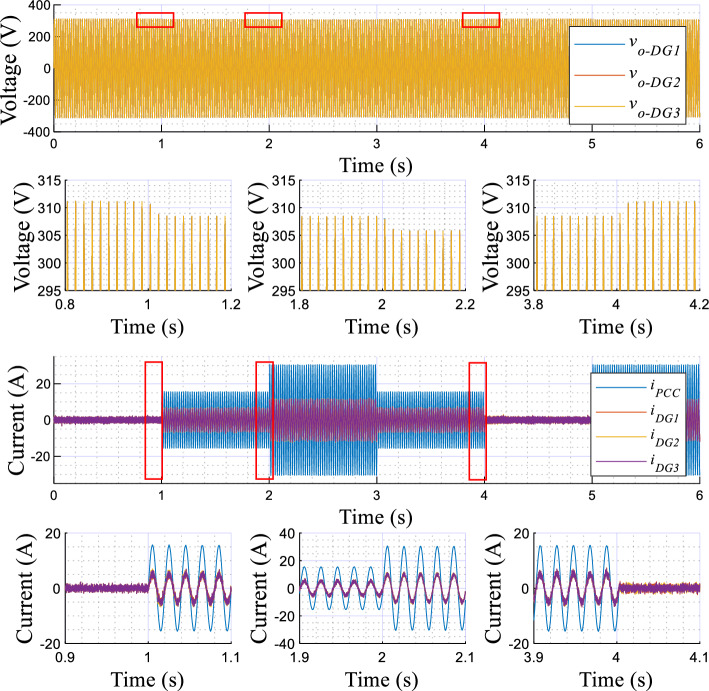
Simulation results showing the output voltage and current of the DG units, with zooms, upon changes in the linear load.

**Figure 11:**
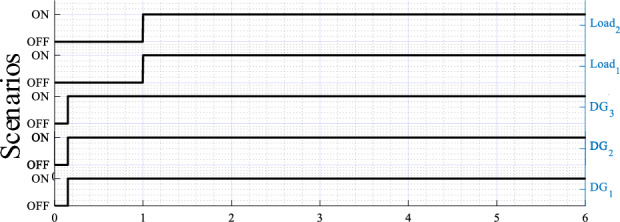
Plots of the applied scenarios in test 2.

**Figure 12:**
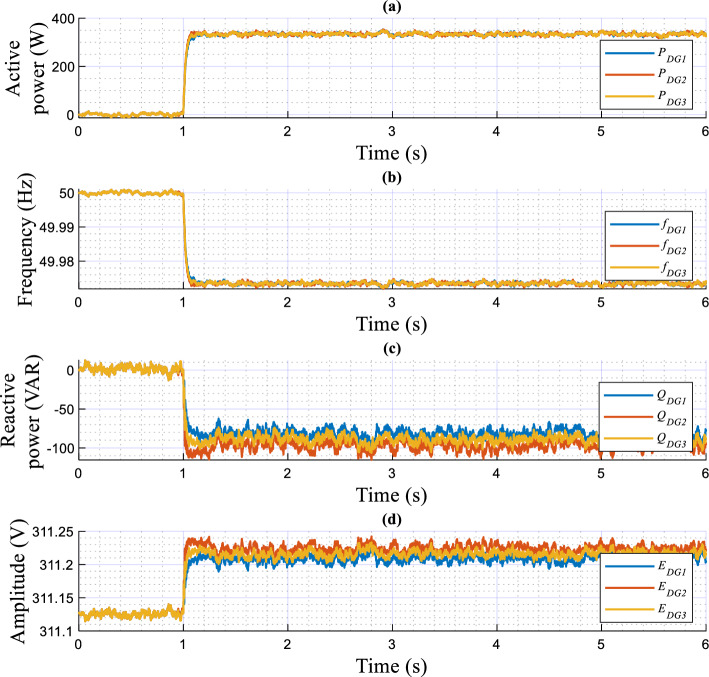
Simulation results of the proposed control strategy in response to sharing nonlinear load.

**Figure 13:**
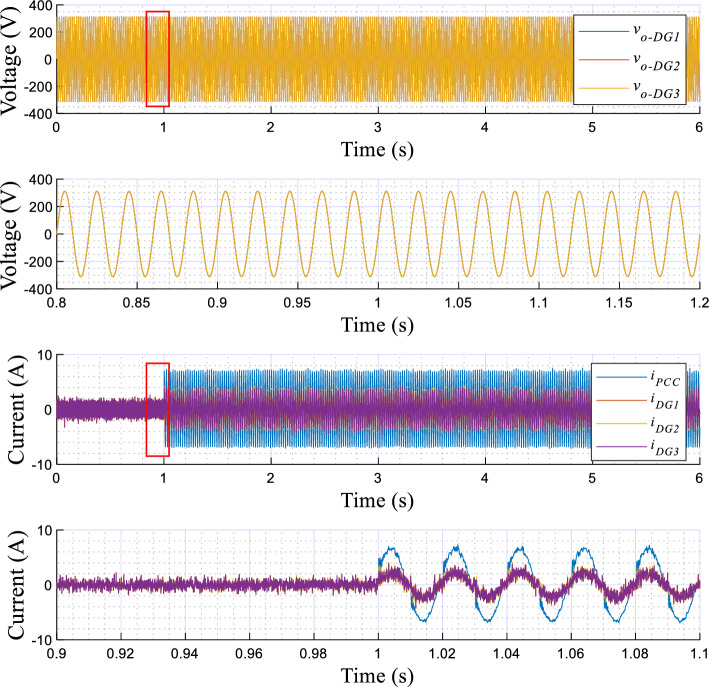
Simulation results of the proposed control strategy in response to sharing the nonlinear load, DG units’ output voltage and currents with their zooms.

The obtained results are given in Figs. 8, 9, 10, 11, 12, 13. Figure 8 depicts the performed scenarios for the linear loads 1 and 2 connection and disconnection. While Figs. 9 and 10 display the simulation results in response to changes in the linear load. These figures portray the real and reactive powers, output voltages frequency, and amplitude, and the output voltages and currents sinusoidal plots of the three DGs.

At first time, the MG operates at no load, the frequencies and amplitudes of the inverters are identical and set to their nominal values (*f*_*DG1*_ = *f*_*DG2*_ = *f*_*DG3*_ = *f*_*n*_, and *E*_*DG1*_ = *E*_*DG2*_ = *E*_*DG3*_ = *E*_*n*_), while the active and reactive powers, *P*_*DG1*_, *P*_*DG2*_, *P*_*DG3*_, *Q*_*DG1*_, *Q*_*DG2*_, and *Q*_*DG3*_ are at zero level. Next, when connecting the first linear load at t = 1 s, as shown in Fig. 9, the inverters' frequencies (*f*_*DG1*_, *f*_*DG2*_, and *f*_*DG3*_) and amplitudes (*E*_*DG1*_, *E*_*DG2*_, and *E*_*DG3*_) droop, meanwhile the real and reactive powers rise. At t = 2 s, when connecting the second linear load, the real and reactive powers smoothly increase with a favorable response time, of about 0.02 s, and without any overshoots. Also, the inverters’ frequencies, *f*_*DG1*_, *f*_*DG2*_, and *f*_*DG3*_, and the amplitudes, *E*_*DG1*_, *E*_*DG2*_, and *E*_*DG3*_, exhibit further droop with the same value, and with a faster transient response. Moreover, it can be seen that an accurate sharing of the real power between the three VSIs is achieved. Similar remarks can be considered for the remaining scenarios, upon disconnecting the second and the first loads.

According to Fig. 10, it can be observed that the output voltages of the three inverters, i.e., *v*_*o−DG1*_,* v*_*o−DG2*_, and *v*_*o−DG3*_, are overlapped, and the currents, *i*_*-DG1*_, *i*_*-DG2*_, *i*_*-DG3*_, as well. Also, these variables have pure sinusoidal waveforms and vary with good dynamic response during load changes.

The simulation results illustrating the performance of the proposed controller under nonlinear load conditions are portrayed in Figs. 12 and 13, while Fig. 11 depicts the established scenarios. Figures 12 and 13 show the waveforms of the same variables presented in test 1. Similarly, the MG initiates its operation with a load-free state, then, a nonlinear load is associated at t = 1 s. As noted in Fig. 12, the VSIs' real powers increase and are overlapped, which means that the inverters share equally the active power demand of the load. In addition, these powers reach the load demand rated values, with a short settling time, about 0.02 s, and without any overshoots. Further, in steady-state, there are no oscillating components or ripples. Moreover, this figure illustrates that *f*_*DG1*_, *f*_*DG2*_, and *f*_*DG3*_ droop with identical amounts, and exhibit proper transient response, a settling time of 0.02 s, and no ripples appear in steady-state. Regarding the amplitudes, it can be noticed that they rise to compensate for the load reactive power, which is equally shared among the three VSIs. Based on the output voltages and currents, depicted in Fig. 13, it can be observed that the VSIs' voltages, *v*_*o-DG1*_, *v*_*o-DG2*_, and *v*_*o-DG3*_, are overlapped and have pure sinusoidal waveforms, while the currents are, also, matched and take the form of the nonlinear load current. Further, it appears that dynamic responses with good performance are achieved.

**Figure 14:**
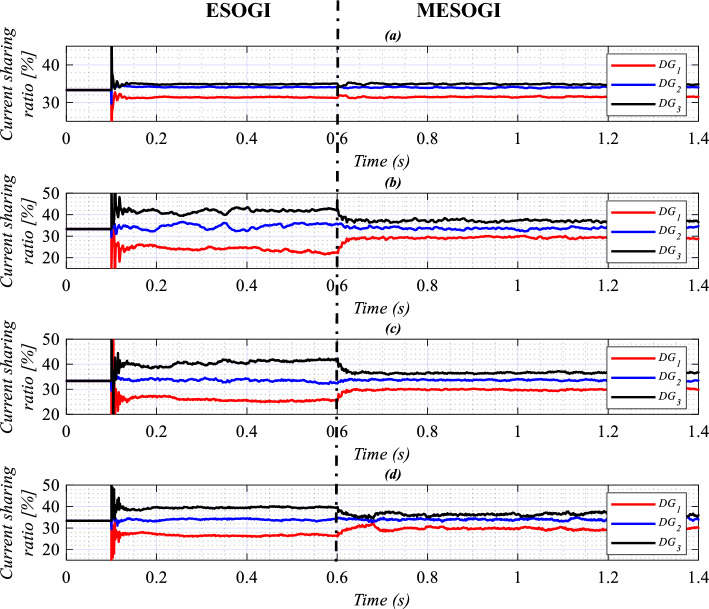
The performance of the proposed virtual impedance compared to the one implemented using ESOGI-based power-sharing controls under nonlinear load conditions.

Figure 14 displays the plots of the current sharing ratio of each DG unit corresponding to the 1st (**a**), 3rd (**b**), 5th (**c**), and 7th (**d**) harmonics, computed using (24), given below. In this figure, the performance of the proposed virtual impedance-based power-sharing control scheme is compared to the one implemented using ESOGI. From Fig. 14a, it can be seen that the ratio of the current shared among VSIs at the fundamental frequency is the same (about 100/3%) for both the ESOGI and MESOGI-based control schemes. While, from Fig. 14b–d, it can be noticed that the current sharing ratio at the 3rd, 5th, and 7th harmonics is changed to be closer to 100/3% when applying the MESOGI-based control strategy at t = 0.6 s. This means that the current circulated among the VSIs is reduced, which can contribute to improving harmonic current sharing.

24 $$di_{hi,j} = \left| {\frac{{i_{hi.j} }}{{i_{hi.DG1} + i_{hi.DG2} + i_{hi.DG3} }}} \right| \times 100$$

where $$i = 1,_{{}} 3,_{{}} 5,_{{}} 7$$ and $$j = DG1,_{{}} DG2,_{{}} DG3$$.

## Experimental results

**Figure 15:**
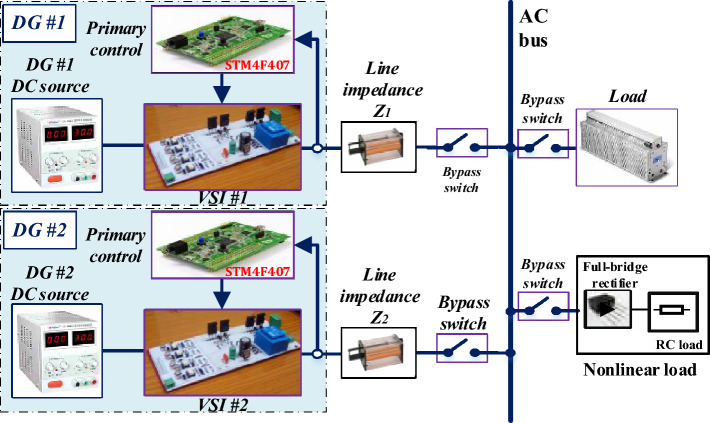
The experimental setup of two paralleled VSIs.

In order to validate the effectiveness of the proposed control approach, an experimental setup of an islanded microgrid is conducted, as shown in Fig. 15. In this system, the control algorithm is implemented using the STMicroelectronics STM32F407VGT6 *µC*, operating at a switching frequency of 10 kHz. Also, the control algorithm of each inverter runs on a dedicated microcontroller, without any communication between them. Table 2 contains the key parameters that are utilized in the practical tests. In addition, the tests and scenarios under consideration are similar to those of the simulation case studies, except for different times. Note that the linear load is presented by a resistive load with a value of 20 Ω, while the nonlinear load is modeled by a full-bridge diode rectifier fed an RC load (R = 20 Ω, C = 470 μF).

**Table 2:** mg system and control parameters.

Parameters	Symbol	Unit	Value
Nominal voltage (RMS)	*E*_*n*_	V	24
Nominal frequency	*f*_*n*_	Hz	50
Switching frequency	*f*_*s*_	kHz	10
DC source voltage	*U*_*DC*_	V	32
Capacitor of the output filter	*C*	µF	26
Inductor of the output filter	*L*	mH	2.7
DG #1 line impedance	*L*_*1*_	mH	0.5
DG #2 line impedance	*L*_*2*_	mH	0.8
Virtual inductance	*L*_*v*_	mH	4
Virtual Resistance	*R*_*v*_	Ω	1
*P/ω* droop gain	*m*	rad/(W. s)	0.0003
*Q/V* droop gain	*n*	V/Var	0.003
P gain of the voltage controller	*k*_*pv*_	µF.rad/s	0.1307
I gain of the voltage controller	*k*_*pi*_	mH.rad/s	32.5476
P gain of the current controller	*k*_*iv*_	mH.rad/s	146 × 10^5^
I gain of the current controller	*k*_*ii*_	mH.rad/s	1.02 × 10^5^

**Figure 16:**
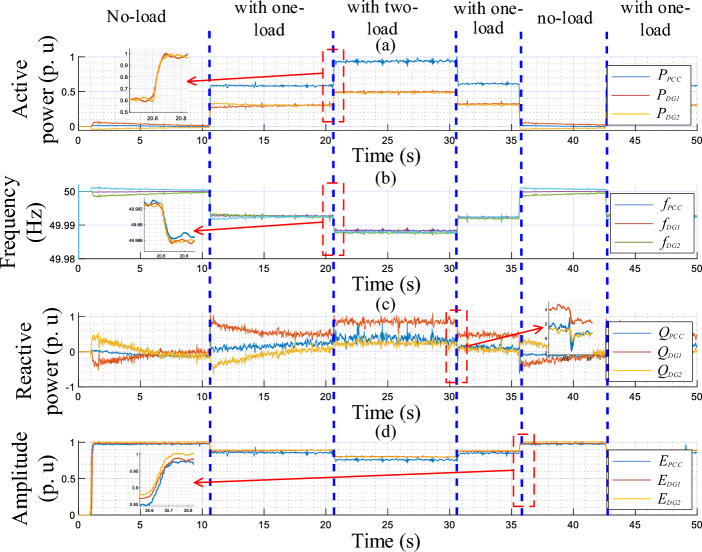
Performance of the proposed control strategy in response to linear load change.

**Figure 17:**
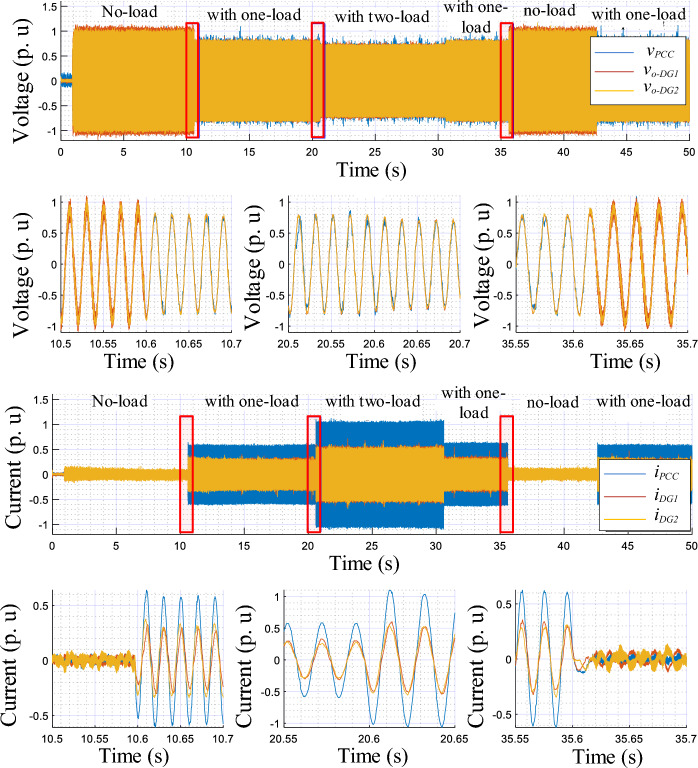
Time evolution of the inverters’ output voltage and current, and at the AC bus with zooms, in response to linear load variation.

The experimental results upon change in the linear load are presented in Figs. 16 and 17, portraying the dynamic response of the real and reactive powers, amplitudes, frequencies, output currents, and output voltages of both the load and VSIs. These figures reveal the following observations:

- In a no-load operation, both powers and output currents are null, while *f*_*DG1*_, *f*_*DG2*_, *E*_*DG1*_, and *E*_*DG2*_ are set to their nominal values.
- When the linear loads are connected and disconnected, the real and reactive powers are accurately shared among the VSIs, showing favorable transient responses, fast response time with no overshoots (see zooms), and no ripples at a steady state.
- The output voltage frequencies *f*_*DG1*_, *f*_*DG2*_, *E*_*DG1*_, and amplitudes *E*_*DG2*_ drop and grow with the same amounts, during the load changes
- Pure sinusoidal waveforms of the DGs’ output voltages and current are achieved.

**Figure 18:**
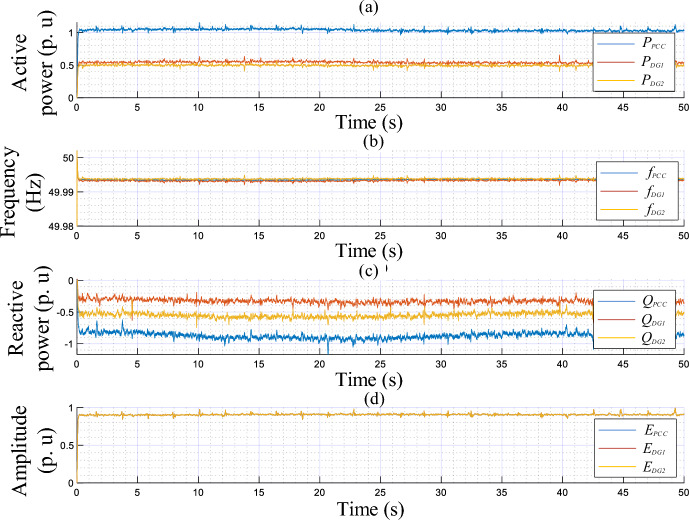
Performance of the proposed control strategy for the case of supplying nonlinear load.

**Figure 19:**
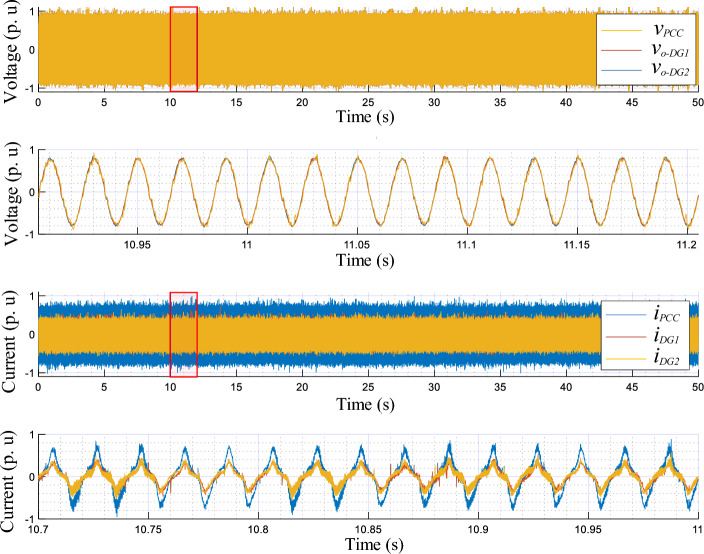
Time evolution of the inverters’ output voltage and current, and at the AC bus, with zooms, in response to nonlinear load sharing operation.

Figures 18 and 19 highlight the experimental results of the proposed control strategy for the case of sharing a nonlinear load and depict the same variables of the first test. Based on these figures, when the nonlinear load connects at t = 0.75 s, one can conduct that:

- The VSIs share accurately the active power delivered to the nonlinear load.
- The inverters’ frequencies, *f*_*DG1*_ and *f*_*DG2*_, droop with identical amounts corresponding to the load demand.
- The same effect is considered for the VSIs’ reactive powers and amplitudes.
- The output voltages and currents of both inverters are overlapped, meanwhile, the currents match the load current’s distorted shape.

## Conclusion

Aiming to achieve accuracy in reactive and harmonic power-sharing among droop-controlled parallelized VSIs in an islanded MG, an improved virtual impedance-based power-sharing control scheme was proposed. The virtual impedance is implemented based on a MESOGI method suitable for harmonics and DC offset estimation/rejection, which can provide an accurate estimation of the quadrature harmonic components of a single-phase signal free from DC offset at selected frequencies. This proposal has made the implemented virtual impedance immune to DC and taken into consideration the harmonic current. Therefore, the designed control scheme based on the implemented virtual impedance was able to alleviate the effect of the line impedance mismatches, thereby enhancing power-sharing. The main focus was given to the derivation of the mathematical model of the MESOGI. This model was used to obtain the mathematical formulation of the implemented virtual impedance based on MESOGI. Then, a model of the equivalent inverter impendence including the implemented virtual impedance and inner control dynamics was derived; and based on it; the effect of the virtual impedance was studied. The performance of the proposed control scheme is assessed through simulation and experimental tests and further via a comparison simulation study with an implemented virtual impendence based on ESOGI. The findings validate the effectiveness of the proposed control scheme in improving the share of reactive and harmonic power under nonlinear load conditions as well as further enhancement compared to the reported ESOGI method.

## Figures and Tables

**Figure 20:**
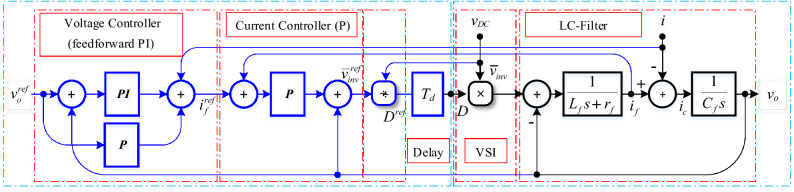
Closed-loop average model of voltage-controlled LC-filtered VSI.

## Data Availability

The datasets used and/or analysed during the current study available from the corresponding author on reasonable request.

## Appendix A

The development that allowed the derivation of the closed-loop transfer functions, *G*_*vo-vo*_ and *z*_*o*_, of the voltage and current control loops are given here.

Based on the closed-loop average model of the double-loop inner controller shown in Fig. 20, which is composed of the model of the system, i.e., LC-filtered VSI, controllers’ functions, and delay unit, and after some mathematical manipulations the following expressions can be obtained:

25 $$\left\{ \begin{gathered} G_{vo - vo} \left( s \right) = \frac{{b_{11} s + b_{10} }}{{a_{4} s^{4} + a_{3} s^{3} + a_{2} s^{2} + a_{1} s + a_{0} }} \hfill \\ z_{o} \left( s \right) = G_{vo - i} \left( s \right) = \frac{{s\left( {b_{22} s^{2} + b_{21} s + b_{20} } \right)}}{{a_{4} s^{4} + a_{3} s^{3} + a_{2} s^{2} + a_{1} s + a_{0} }} \hfill \\ \end{gathered} \right.$$

being *G*_*vo-vo*_ and *z*_*o*_ the transfer functions relating the output voltage to its voltage reference and output current, respectively, while the parameters $a_{0} ,_{{}} a_{1} ,_{{}} a_{2} ,_{{}} a_{3} ,_{{}} a_{4}$ $b_{10} ,b_{11} ,b_{20} ,b_{21}$ and $b_{22}$ are given as follows:

26 $$\left\{ \begin{gathered} a_{0} = k_{P - If} k_{I - E} \hfill \\ a_{1} = k_{P - If} \left[ {k_{P - E} - \frac{{T_{d} }}{2}k_{I - E} } \right] \hfill \\ a_{2} = \left[ {T_{d} - \frac{{T_{d} }}{2}k_{P - If} k_{P - E} + C_{f} \left[ {r_{f} + k_{P - If} } \right]} \right] \hfill \\ a_{3} = C_{f} \left[ {L_{f} + \left( {r_{f} - k_{P - If} } \right)\frac{{T_{d} }}{2}} \right] \hfill \\ a_{4} = \left[ {\frac{{C_{f} L_{f} T_{d} }}{2}} \right] \hfill \\ \end{gathered} \right.,_{{}} \left\{ \begin{gathered} b_{10} = k_{P - If} k_{I - E} \hfill \\ b_{11} = - \frac{{T_{d} }}{2}k_{P - If} k_{I - E} \hfill \\ b_{20} = r_{f} \hfill \\ b_{21} = L_{f} + \frac{{T_{d} }}{2}r_{f} \hfill \\ b_{22} = \frac{{L_{f} T_{d} }}{2} \hfill \\ \end{gathered} \right.$$

where *T*_*d*_ denotes the time delay, *L*_*f*_,* r*_*f*_, and *C*_*f*_ are the LC filter inductor, resistor, and capacitor, *k*_*P-E*_, *k*_*I-E*_* k*_*P-If*_, and *k*_*I-If*_ are the voltage and current regulators proportional and integral gains.

In addition, it is worth mentioning that, in Fig. 20, *D* and *D*^*ref*^ define the delayed and the actual duty cycles, respectively, while $i_{f} ,_{{}} \overline{v}_{inv}^{{}}$, $i_{f}^{ref}$, and $\overline{v}_{inv}^{ref}$ are the actual inductor current and the averaged voltage of the inverter and their references.

## Appendix B

This appendix presents the demonstration of how we determine the ESOGIs’ gain “*k*” corresponding to the harmonics’ estimation. As stated earlier, the value of *k* of the ESOGI units, corresponding to harmonic estimates, is divided by the harmonic component’s order (*n*) to maintain a consistent settling time for all the ESOGI units.

It is worth noting that the settling time, *t*_*s-h1*_, of a second-order transfer function, can be expressed in terms of its damping factor “*k*” and resonance frequency (*ω*_*res*_) for an ESOGI unit respective to the fundamental component estimate, as follows:

27 $$t_{s - h1} = \frac{4}{{\frac{k}{2}_{{}} \omega_{res - h1} }}$$

Hence, the mathematical formulation of the settling time, *t*_*s-hn*_, corresponding to the *n*-order harmonic estimation can be defined as:

28 $$t_{s - hn} = \frac{8}{{k_{hn} \omega_{res - hn} }} = \frac{8}{{k_{hn} \left( {n \times \omega_{res - h1} } \right)}}$$

where *ω*_*res-hn*_ stands for the ESOGI’s resonance frequency corresponding to the *n*-order harmonic estimation.

In order to preserve an identical settling time as that of the dynamic estimation of the fundamental component, i.e., *t*_*s-hn*_ = *t*_*s-h1*_, the expression for *k*_*hn*_ for each unit should be defined as follows:

29 $$k_{hn} = \frac{k}{n}$$

## Appendix C

]This appendix provides the expressions of the closed-loop transfer functions,$G_{BF.\alpha - 1,3,5,7}$, $G_{BF.\beta - 1,3,5,7}$, and $G_{BF.DC}$, which define the relation between the MESOGI’s output current components; the fundamental direct and quadrature components, and the DC component; with the actual input current. The expressions of $G_{BF.\alpha - 1,3,5,7}$, $G_{BF.\beta - 1,3,5,7}$, and $G_{BF.DC}$ are:

30 $$\begin{aligned} G_{{BF.\alpha - 1}} & = \frac{{k\omega _{{}} s^{7} + 83k\omega ^{3} _{{}} s^{5} + 1891k\omega ^{5} _{{}} s^{3} + 11025k\omega ^{7} s}}{{Den(1)}} \\ G_{{BF.\alpha - 3}} & = \frac{{k\omega _{{}} s^{7} + 75k\omega ^{3} _{{}} s^{5} + 1299k\omega ^{5} _{{}} s^{3} + 1225k\omega ^{7} s}}{{Den(1)}} \\ G_{{BF.\alpha - 5}} & = \frac{{k\omega _{{}} s^{7} + 59k\omega ^{3} _{{}} s^{5} + 499k\omega ^{5} _{{}} s^{3} + 441k\omega ^{7} s}}{{Den(1)}} \\ G_{{BF.\alpha - 7}} & = \frac{{k\omega _{{}} s^{7} + 35k\omega ^{3} _{{}} s^{5} + 259k\omega ^{5} _{{}} s^{3} + 225k\omega ^{7} s}}{{Den(1)}} \\ G_{{BF.\beta - 1}} & = \frac{{k\omega ^{2} _{{}} s^{6} + 83k\omega ^{4} _{{}} s^{4} + 1891k\omega ^{6} _{{}} s^{2} + 11025k\omega ^{8} }}{{Den(1)}} \\ G_{{BF.\beta - 3}} & = \frac{{3k\omega ^{2} _{{}} s^{6} + 225k\omega ^{4} _{{}} s^{4} + 3897k\omega ^{6} _{{}} s^{2} + 3675k\omega ^{8} }}{{Den(1)}} \\ G_{{BF.\beta - 5}} & = \frac{{5k\omega ^{2} _{{}} s^{6} + 295k\omega ^{4} _{{}} s^{4} + 2495k\omega ^{6} _{{}} s^{2} + 2205k\omega ^{8} }}{{Den(1)}} \\ G_{{BF.\beta - 7}} & = \frac{{7k\omega ^{2} _{{}} s^{6} + 245k\omega ^{4} _{{}} s^{4} + 1813k\omega ^{6} _{{}} s^{2} + 1575k\omega ^{8} }}{{Den(1)}} \\ G_{{BF.DC}} & = \frac{{\omega _{f} s^{8} + 84\omega ^{2} \omega _{f} s^{6} + 1974\omega ^{4} \omega _{f} s^{4} + 12916\omega ^{6} \omega _{f} s^{2} + 11025\omega ^{8} \omega _{f} }}{{Den(2)}} \\ \end{aligned}$$

where

31 $$Den(1) = s^{8} + 4k\omega_{{}} s^{7} + 84\omega^{2}_{{}} s^{6} + 252k\omega^{3}_{{}} s^{5} + 1974k\omega^{4}_{{}} s^{4} + 3948k\omega^{5}_{{}} s^{3} + 12916\omega^{6}_{{}} s^{2} + 12916k\omega^{7}_{{}} s + 11025\omega^{8}$$

32 $$\begin{aligned} Den(2) & = s^{9} + \left( {\omega_{f} + 4k\omega } \right)s^{8} + \left( {84\omega^{2} + 4k\omega \omega_{f} } \right)s^{7} + \left( {252k\omega^{3} + 84\omega^{2} \omega_{f} } \right)s^{6} + \left( {1974\omega^{4} + 252k\omega^{3} \omega_{f} } \right)s^{5} \\ & \;\; + \left( {3948k\omega^{5} + 1974\omega^{4} \omega_{f} } \right)s^{4} + \left( {12916\omega^{6} + 3948k\omega^{5} \omega_{f} } \right)s^{3} + 12916\left( {k\omega^{7} + \omega^{6} \omega_{f} } \right)s^{2} \\ & \;\; + \left( {11025\omega^{8} + 12916k\omega^{6} \omega_{f} } \right)s + 11025\omega^{8} \omega_{f} \\ \end{aligned}$$
